# Sparse Decomposition of Heart Rate Using a Bernoulli-Gaussian Model: Application to Sleep Apnoea Detection

**DOI:** 10.3390/s23073743

**Published:** 2023-04-04

**Authors:** Bruno H. Muller, Régis Lengellé

**Affiliations:** 1 Pharma Partnering in Research & Strategy (PPRS), 68000 Colmar, France; 2 LIST3N, Université de Technologie de Troyes (UTT), 10004 Troyes, France

**Keywords:** heart rate, sparse decomposition, sleep apnoea, cardiac events

## Abstract

In this paper, we propose a sparse decomposition of the heart rate during sleep with an application to apnoea–RERA detection. We observed that the tachycardia following an apnoea event has a quasi-deterministic shape with a random amplitude. Accordingly, we model the apnoea-perturbed heart rate as a Bernoulli–Gaussian (BG) process convolved with a deterministic reference signal that allows the identification of tachycardia and bradycardia events. The problem of determining the BG series indicating the presence or absence of an event and estimating its amplitude is a deconvolution problem for which sparsity is imposed. This allows an almost syntactic representation of the heart rate on which simple detection algorithms are applied.

## 1. Introduction

Apnoeas correspond to periods of no (apnoea) or shallow (hypopnoea) breathing during sleep. Another type of event is respiratory effort-related arousal (RERA), which causes a drop in oxygen saturation without qualifying as apnoea or hypopnoea. The gold standard for sleep apnoea detection is polysomnography (PSG), for which electroencephalogram (EEG), electrooculogram (EOG), electromyogram (EMG), electrocardiogram (ECG), respiratory signals (airflow, effort), and oxygen saturation are recorded.

Due to the significant impact of a high number of apnoeas during sleep on daytime sleepiness and health problems (such as accidents, memory loss, depression, diabetes, cardiovascular problems, hypertension, decreased cognitive skills, neurological issues, and liver problems [1]), a treatment known as continuous positive airway pressure was proposed in the 1980s [2].

However, PSG is expensive, inconvenient, and must be conducted in a medical facility. This explains the search for surrogate signals, which are much easier to record and allow for apnoea detection in a friendlier environment. It is well known that apnoea impacts the heart rate. During an apnoea event, the heart rate decreases (relative bradycardia). After the apnoea event, respiration starts again and an increase in heart rate (tachycardia) is observed [3].

The heart rate, which can be measured using electrocardiography (ECG) or photoplethysmography (PPG), may allow for the reconstruction of the respiratory signal [4,5]. However, this solution requires knowledge of the entire ECG or PPG signal. In our application, our target device, Somno-Art (https://www.somno-art.com, accessed on 2 November 2022), provides a time series of RR intervals, from which we reconstruct the HR signal (which is regularly sampled at 1 Hz) [6].

The Somno-Art device and its associated software are primarily intended for automated sleep stage analysis using recorded movements and pulse rate. In [7], the authors demonstrated an underestimation of sleep stage N3 duration for patients with obstructive apnoea syndrome. This observation motivated the study of an apnoea detection algorithm using only the heart rate and movement signals to improve sleep detection by reducing ambiguities caused by apnoea, and as a screening tool.

Although our final objective is to use the method presented here as an add-on to Somno-Art, we used signals extracted from PSG along with reference sleep and apnoea scoring.

Literature studies on apnoea detection using electrophysiological signals (e.g. ECG, pulse oximetry, respiration, sound) or heart rate variability (HRV) are abundant; moreover, surveys exist [8,9,10]. The methods are mostly based on feature extraction, classification and, more recently, deep learning methods.

In this paper, we propose a signal-processing approach that has never been used for apnoea detection using heart rate; it appears to be very well adapted and provides a good description of apnoea–hypopnoea–RERA events. The idea is to model the heart rate as the convolution of a reference signal and a Bernoulli–Gaussian (BG) time series. Given an observed heart rate signal and the reference signal, we propose to perform deconvolution to estimate the BG time series. Obtaining the BG series by deconvolution is very similar to the sparse decomposition of a signal (compressed sensing). Deconvolution is an ill-posed problem, so we constrain the solution to be sparse. This last property is characteristic of a BG process, which makes it a good match for the sparseness constraint used. As explained in the paper, the reference signal is selected to describe either tachycardia or bradycardia events.

This paper is organised as follows: In Section 2, we provide an overview of sparse methods and the important role of the norm-based constraint. We connect the optimisation problem to that of sparse deconvolution using a Bernoulli–Gaussian model. This section begins with a quick summary of sparse decomposition methods (Section 2.1); we introduce the probabilistic model of the observed signal (Section 2.2) and the resulting apnoea detection model in Section 2.3. The objective, the data used to implement the model, and additional data for testing are presented in Section 2.4. Finally, the results of our data are presented in Section 3.1; the application of this method to external data (PhysioNet Apnea ECG database) is presented in Section 3.2. We present a short discussion in Section 4 and our conclusions and perspectives in Section 5.

## 2. Materials and Methods

### 2.1. Sparse Decomposition

Sparse models are models where only a reduced number of parameters play an important role. Sparse representations have found many applications in signal and image processing, including computer vision, machine learning, array processing, data mining, and system identification [11,12,13,14,15,16,17,18,19,20,21,22,23,24]. An extensive survey of sparse representations can be found in [25]. In linear regression, for example, the objective is to predict an output vector *y* (N×1) using a data matrix *X* (N×p) where *N* is the number of equations and *p* is the number of predictors. This is N>p for our application, but methods have also been considered in the underdetermined case, where p>N. Using the model $y_{i}=\sum_{j=1}^{p}X_{ij}\beta_{j}+e_{i};\;i\in 1,\ldots ,N$ where $e_{i}$ represents an error term, β is the vector of the model parameters to be determined; in matrix form, the least-squares solution is:

$$\beta^{*}=\underset{\beta }{arg min}\;{\left||y-X\beta |\right|}_{2}^{2}$$

where ${\left||.|\right|}_{2}^{2}$ denotes the squared $L_{2}$ (Euclidean) norm. An additional intercept term $\beta_{0}$ might be included in the model. In general, all of the components of β will be non-zero, particularly if the columns of matrix *X* are highly correlated, so the interpretation of β will be challenging. In this case, we need to constrain the solution (or regularise the problem) in such a way that the solution makes use of a limited number of predictors. Of course, this will be achieved at the cost of an increase in the error, but this extra cost might be compensated, for example, by making the solution interpretable (this will be illustrated in Remark 1 in the following section). The first attempt to find a sparse solution (and deal with multicollinearity among predictors) is ridge regression, which involves adding a penalty term to the problem:

$$\beta^{*}=\underset{\beta }{arg min}\;{\left||y-X\beta |\right|}_{2}^{2}+{\lambda ||\beta ||}_{2}^{2}$$

where λ≥0 allows for a trade-off between the error term ${\left||y-X\beta |\right|}_{2}^{2}$ and the penalization term ${\left||\beta |\right|}_{2}^{2}$. Using Lagrangian duality, this problem can be written as the following constrained optimisation problem:

$$\begin{array}{c} \min_{\beta }{\left||y-X\beta |\right|}_{2}^{2}\\ {s.t.\;||\beta ||}_{2}^{2}\leq \tau \end{array}$$

**Figure 1:**
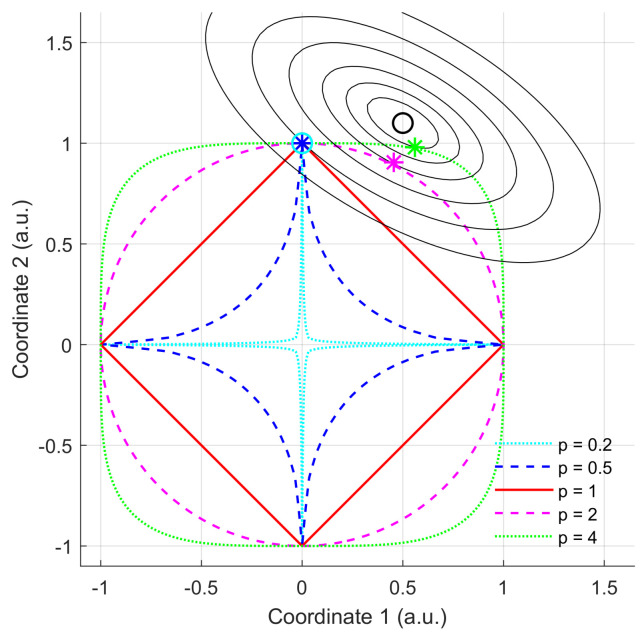
Minimisation of a quadratic function under different $L_{p}$ norm inequality constraints (detailed in the text above).

However, making some of the components of β exactly zero is not always desirable with the ridge regression method. This is the reason why the least absolute shrinkage and selection operator (LASSO) method was introduced in 1996 by R. Tibshirani [26]. The LASSO method consists of solving the following optimisation problem:

$$\begin{array}{c} \min_{\beta }{\left||y-X\beta |\right|}_{2}^{2}\\ {s.t.\;||\beta ||}_{1}\leq \tau \end{array}$$

where the constraint now uses the $L_{1}$ norm of β, whose influence on the sparseness of the solution is described in Figure 1.

This figure depicts the contours of a bivariate quadratic function (black ellipses) with the global minimum represented by the black symbol ‘o’. The constraint is defined by the $L_{p}$ norm for different values of *p*. The feasible domain corresponds to the interior of the domain, defined by the outer dotted green curve (p=4) and outer dashed magenta curve (p=2), which correspond to the Euclidean norm. The red plain curve corresponds to (p=1), the inner dashed blue curve corresponds to (p=0.5), and the inner dotted cyan curve corresponds to (p=0.2). The solutions are indicated by the coloured symbols located in the domain boundary corresponding to each selected value of *p*. As can be seen in this figure, when p≤1, the solution becomes sparse (only one component of the solution differs from 0).

The price to pay is a higher value of the function to be minimised. It is important to notice that, when p<1, the feasible domain is no longer convex, which impacts Lagrangian duality and leads to local minima of the function. For any problem of this kind (minimising a function under an $L_{p}$ norm constraint), the maximally sparse solution, if attainable, will be obtained using the $L_{0}$ pseudo-norm: it provides the number of non-zero elements of a vector. However, using this pseudo-norm leads to a combinatorial NP-hard optimisation problem [27,28].

Different approaches have been proposed to find an approximate solution of

(1) $$\begin{array}{c} \min_{\beta }{\left||y-X\beta |\right|}_{2}^{2}\\ {s.t.\;||\beta ||}_{0}\leq k \end{array}$$

or of the (similar) problem:

(2) $$\min_{\beta }{\left||y-X\beta |\right|}_{2}^{2}+{\lambda ||\beta ||}_{0}$$

or, in the noiseless case:

(3) $$\begin{array}{c} \min_{\beta }{\left||\beta |\right|}_{0}\\ s.t.\;X\beta =y \end{array}$$

which are NP-hard. Among others, one strategy is to approximate the $L_{0}$ norm by a differentiable version [29,30,31,32]. Another possibility, widely explored, is to use a greedy strategy in the spirit of matching pursuit [33], initially proposed in the underdetermined case, p>N (sparse coding), which evolved to orthogonal matching pursuit (OMP) and orthogonal least squares (OLS). While computationally efficient, OMP and OLS do not perform well when the columns of *X* are highly correlated.

### 2.2. Probabilistic Modelling of the Observed Signal and Connection to $L_{2}-L_{0}$ Optimisation

**Figure 2:**
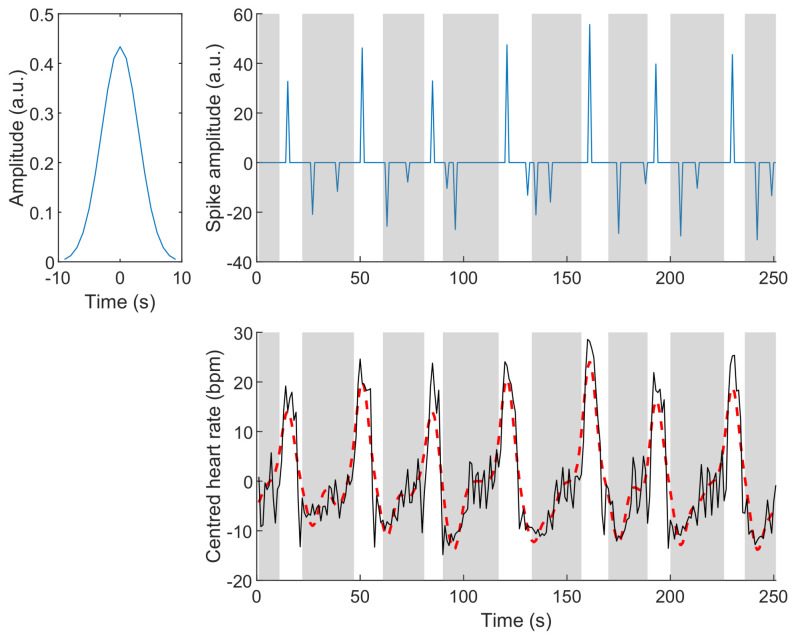
(**Upper left**): reference signal; (**upper right**): sparse model of the heart rate; (**lower right**): high-pass filtered heart rate during a series of consecutive apnoeas (plain black line) and reconstructed heart rate (dashed red line) using the reference signal and the series of spikes. Apnoeas are highlighted by the grey patches.

To illustrate our approach, we consider a time interval where the heart rate is clearly impacted by apnoea (lower right panel in Figure 2). In this figure, the high-pass filtered heart rate is represented by the solid black curve. This is our observed signal *y*. The dashed red signal represents a model of the heart rate obtained by the convolution of a reference signal (upper left panel) and the series of spikes (upper right panel). In both right panels, apnoea (scored using respiratory and SpO_2_ signals) is highlighted by the grey patches. The reference signal (upper left panel) was chosen to describe tachycardia. Using this reference, bradycardia, which is generally longer than tachycardia, is modelled as one or more consecutive negative reference signals. As can be seen, the analysis of the sparse series of spikes (Figure 2) provides an interesting description of the behavior of the heart rate during apnoea: the first negative spike approximately corresponds to the beginning of the bradycardia, and the following positive spike indicates the tachycardia.

As our model results from the convolution of the reference signal with the series of spikes, obtaining the series of spikes from the heart rate can be considered a deconvolution problem in which sparseness of the solution is required. The noisy convolution model can be written in matrix form

y=Xβ+e

where every column (element of our dictionary) of matrix *X* corresponds to a time-shifted version of the reference signal and *e* is the error vector between the observed signal y and the model output Xβ. The fact that the dictionary elements (columns of the matrix *X*) are shifted versions of the same reference signal results in a Toeplitz structure of *X*.

**Figure 3:**
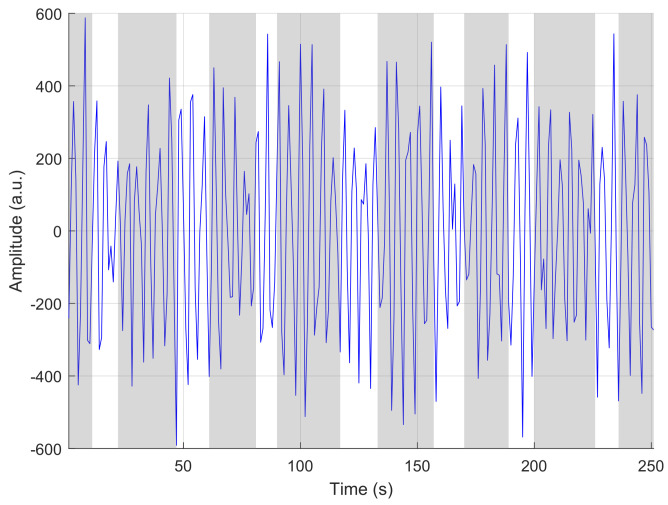
Non-sparse solution obtained by minimization of $||y-{X\beta ||}_{2}^{2}$ w.r.t β (without penalisation) in the same segment of the signal as in Figure 2; the greyed areas correspond to ground-truth apnoea.

**Remark** **1.** *Comparing the sparse series (upper right plot in Figure 2) to the non-penalised least-squares solution shown in Figure 3 shows the dramatic improvement of interpretability brought by the search for a sparse solution, while the least-squares solution leads to a smaller quadratic error ${\left||e|\right|}_{2}^{2}$ compared to that obtained with the sparse model.*

Our problem is to minimise the reconstruction error energy, i.e., to minimise w.r.t. β: $||y-{X\beta ||}_{2}^{2}$, given *X* and *y*, under the constraint that β is sparse, which is Problem (1) if sparseness is defined by the $L_{0}$ norm of the solution. In our case, an exhaustive search for the sparse solution is intractable since we have to explore about $2^{30000}$ solutions.

We now introduce a probabilistic model of the series of spikes. This series can be easily modelled by a Bernoulli–Gaussian (BG) process. Bernoulli variables, which are supposed to be independent and identically distributed (iid), are associated with the position of spikes (non-zero elements of β that define the active set: the selected columns of *X*), while amplitudes are supposed to be iid-Gaussian and independent from the Bernoulli variables. Sparse deconvolution under the Bernoulli–Gaussian assumption has already been studied in the literature throughout the 1990s, in particular in [34,35], whose works are based on [36,37,38,39,40,41,42]. We shall make the possible connection between sparse deconvolution and the resolution of Problem (2) in the following.

A BG process β is defined as a random vector associated with a Bernoulli random vector $q\;(q_{i}\in \left\{0,\;1\right\};\;i\in \left\{1,\ldots ,p\right\})$ defining the active set (the selected columns of matrix *X*), and a Gaussian random vector $r∼N(0,\;\sigma_{\beta }^{2}\;I_{p})$, such that $\beta_{i}=q_{i}r_{i}$. Under the previous assumptions, the likelihood of (q,r) is given by:

$$l\left(q,r\right)=l\left(q\right)l\left(r\right)=\rho^{\left(||q|{|}_{0}\right)}{\left(1-\rho \right)}^{\left(p-||q|{|}_{0}\right)}G\left(r,\;\sigma_{\beta }^{2}I_{p}\right)$$

where ρ denotes the probability $p(q_{i}=1)\;\forall i$, G(·,C) is the zero-mean multidimensional Gaussian probability density function with covariance matrix *C*. If the error *e* is assumed iid, zero-mean Gaussian with variance $\sigma_{e}^{2}$, and independent from β, the posterior likelihood l(q,r|y) becomes [43]:

$$l\left(q,r|y\right)\propto G\left(y-Xr,\sigma_{e}^{2}I_{N}\right)\rho^{\left(||q|{|}_{0}\right)}{\left(1-\rho \right)}^{\left(p-||q|{|}_{0}\right)}G\left(r,\;\sigma_{\beta }^{2}I_{p}\right)$$

where ∝ denotes proportionality.

Denoting *t* as the vector, such that r=(t,u) with $u=\{r_{i}:q_{i}=0\}$, it has been shown [43] that:

$$-2\sigma_{e}^{2}\log\left(l\left(q,r|y\right)\right)=||y-X_{T}{t||}_{2}^{2}+\frac{\left(\sigma_{e}^{2}\right)}{\left(\sigma_{\beta }^{2}\right)}{\left||t|\right|}_{2}^{2}+2\sigma_{e}^{2}\log\left(\frac{1-\rho }{\rho }\right){\left||q|\right|}_{0}$$

where $X_{T}$ represents the matrix composed of *active* columns of $X\;(T=\left\{i:\;q_{i}=1\right\})$. As pointed out in [43], the weight of $q_{0}$ is non-negative if $\rho \leq \frac{1}{2}$, which is coherent with the sparseness assumption. In the same paper [43], authors considered the limit of this expression when $\rho_{\beta }^{2}⟶+\infty$ (since sparseness only refers to ${\left||q|\right|}_{0}$). This also allows us to avoid considering a Gaussian distribution for the amplitudes of the spikes and probabilistic assumptions about the error. Accordingly, the optimisation problem becomes:

$$\min_{q,t}:||y-X_{T}{t||}_{2}^{2}+{\lambda ||q||}_{0}$$

where $\lambda =2\sigma_{e}^{2}\log\left(\frac{1-\rho }{\rho }\right)$, which is shown to be equivalent to

(4) $$\min_{\beta }:||y-{X\beta ||}_{2}^{2}+{\lambda ||\beta ||}_{0}$$

Authors in [43] proposed an efficient algorithm, called the single best replacement (SBR), to iteratively find an approximate solution to Problem (4), in a finite number of iterations, and avoiding, at each iteration, the multiple inversions of $X_{T^{\prime }}^{t}X_{T^{\prime }}$, where $T^{\prime }$ represents any new possible active set when iteratively adding (respectively, removing) one non-null component to (respectively, from) T. Their algorithm is summarised in Table II of [44] and was used in this work. It is able to find an approximate solution to Problem (4), in a reasonable time (a few seconds per recording, using a personal computer), with typical values of *N* considered here (N≈30,000). Once the reference signal is selected, the only hyperparameter to tune is λ, which controls the sparseness of the solution; that can be selected using, for example, cross-validation.

### 2.3. RDI Estimator

**Figure 4:**
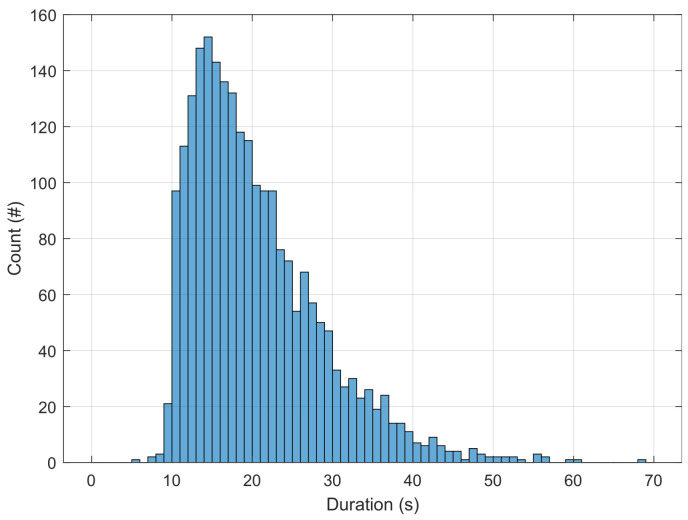
Histogram of apnoea durations, evaluated on the training set.

**Figure 5:**
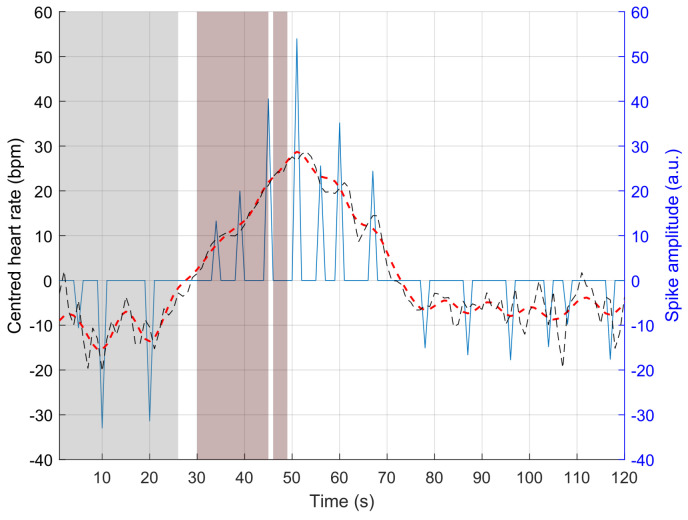
Effect of a movement on the sparse decomposition; sparse model (blue spikes), high-pass filtered heart rate (plain black line), and reconstructed heart rate (dashed red line); apnoea highlighted by the greyed areas and movement highlighted by the red patches.

Using the proposed sparse decomposition method, we have defined a detector that takes into account the temporal organization of the Bernoulli–Gaussian time series and its amplitude (to avoid a possible high rate of false alarms). Our detector is adapted to this temporal organization. As mentioned in the introduction, apnoea corresponds to relative bradycardia followed by tachycardia. The bradycardia event, which appears during the apnoea event, is generally longer (ranging from 5 to 50 s, see Figure 4) than the tachycardia event that appears at the end of the apnoea. Using our sparse representation, apnoea-related bradycardia is associated with a succession of negative spikes, while the following tachycardia event generally corresponds to a single positive spike. Body movements or other cardiac arousals are associated with a relatively long tachycardia, resulting in a succession of positive spikes (see Figure 5). Therefore, to detect apnoea, we focus on each individual positive spike (to discard body movements or other cardiac arousals) that is preceded by one or more negative spikes appearing within the time window $\left[t_{T}-50,t_{T}-5\right]$, where $t_{T}$ denotes the time instant of the (single) positive spike that exceeds a threshold introduced below. The histogram of apnoea durations established from our internal data (Figure 4), which is presented in the following section, helped us define this time window.

We need to select a threshold for each night, computed from the observed heart rate itself, to account for the high inter-individual variability of heart rate, which is known to be strongly influenced by factors such as age, gender, health condition, and other unmeasurable factors.

We would like to remind the reader that the device to be used (Somno-Art) and the associated software were initially developed for automatic sleep staging [6,7] using the heart rate obtained via photoplethysmography and the movements measured by a three-dimensional accelerometer. To select our threshold (i.e., the amplitude of the tachycardia or positive spikes), several possibilities were studied, including using the hypnogram to find transitions from slow wave sleep to light sleep or wake, as these transitions correspond to a change in the sympathovagal balance (and of the HRV). The second considered possibility was to use the response of the heart rate to movements, and the third was to study the dispersion of the heart rate during wake (here again using the hypnogram).

Regarding internal data, we opted for the third solution. The threshold is defined as $\alpha \sigma_{0}$ where $\sigma_{0}$ is the standard deviation of the heart rate during intra-sleep awakenings and α a coefficient to tune. α was selected on the training set (α=2.5); other parameters, e.g., λ, the reference Gaussian model σ, and ⋯, were selected to maximise the correlation between the ground-truth *RDI* and the estimated one, only on the training set. Considering the small number of parameters to be tuned, we used both the validation and test sets to evaluate the model performances.

Regarding external data (PhysioNet ECG database presented in Section 2.4.3), lacking a hypnogram, we considered all of the single positive spikes.

### 2.4. Objective and Data Inventory

#### 2.4.1. Objective

This research focuses on the estimation of the standardised Respiratory Disturbance Index (*RDI*) [45] and the frequency of respiratory events over sleep duration, defined as:

(5) $$RDI=\frac{\left(\#Apnoea+\#Hypopnoea+\#RERAs\right)}{Sleep\;\;duration\;[hours]}$$

where #Apnoea denotes the number of apnoeas during the night, #Hypopnoea the number of hypopnoeas, and #RERAs the number of respiratory effort-related arousals (*RERAs*). Another option could have been the estimation of the standardised Apnea–Hypopnea Index (AHI—[45]), which does not take into account *RERAs*. Both factors can be used to assess apnoea severity, i.e, normal for a score below 5, mild between 5 and 15, moderate between 15 and 30, and severe (above 30) [45].

#### 2.4.2. Internal Data

To evaluate the model and its performances in apnoea detection, we relied on 34 recordings (one for each of the 34 subjects), which were divided into learning, validation, and test datasets; the dataset composition is given in terms of apnoea severity in Table 1, and in terms of age, sex, and body mass index (BMI) in Table 2.

The recordings were established during the time allocated to sleep (between lights off—when the subject attempted to sleep, and lights on—the moment the subject woke up for the day) and consist of synchronous recordings of polysomnography (PSG), an accelerometer (ActiSleep+, ActigraphTM LLC, 49 East Chase Street, Pensacola, FL 32502, USA), and a Somno-Art device (see https://www.somno-art.com/, accessed on 2 November 2022); the latter two were placed on the non-dominant forearm.

The PSG comprises an electroencephalogram (EEG) with 28 electrodes placed following the 10–20 international system, an electrooculogram (EOG) with 2 electrodes, an electromyogram (EMG) with 1 electrode placed on the chin, along with an additional ECG. Each signal has a sampling frequency of 256 Hz.

Finally, considering our objective, for each recording we only used:

- Heart rate (HR) at a 1 Hz resampling frequency. This signal was established from electrocardiography data, from which R-peaks were extracted, RR intervals were measured, quality was reviewed, and instantaneous HR was computed, interpolated, and resampled at 1 Hz.
- Accelerometry data, also at 1 Hz and synchronous with the heart rate. These originate from the wrist and help distinguish movement-induced cardiac events.
- Expert-based sleep scoring and the resulting hypnogram, which represents the temporal distribution of sleep stages through the recording in 30-s epochs, were established based on the polysomnography data. Polysomnography was recorded simultaneously with the Somno-Art device, allowing for future evaluation of the latter as a potential tool for apnoea screening.
- Expert-based assessment was used to identify apnoeas in the signal, which served as our ground truth.
- Assessment of limb movements.

In the following, the proposed model was applied to the heart rate to yield a sparse representation and allow for the easy reading of cardiac events. Using the reference hypnogram, an estimation of *RDI* can be computed, allowing for further evaluation.

#### 2.4.3. The PhysioNet Apnea ECG Database

As will be detailed in Section 3.2, we also tested our algorithm on a publicly available database relative to apnoea detection, based on the cardiac signal and the PhysioNet Apnea ECG database [46,47]; both the full ECG and a one-minute-scored apnoea signal are given.

The PhysioNet challenge dataset comprises three categories A, B, and C:

- Class A contains at least 1 h with an apnoea index of 10 or more, and at least 100 min with apnoea during the recording.
- Class B contains at least 1 h with an apnoea index of 5 or more, and between 5 and 99 min with apnoea during the recording.
- Class C contains fewer than 5 min of apnoea during the recording (which is clearly not equivalent to the AHI).

A full description of the data is publicly available online (see https://www.physionet.org/content/apnea-ecg/1.0.0/additional-information.txt, accessed on 10 November 2022). This database also provides the AHI, which allows for evaluating our algorithm on these data.

**Figure 6:**
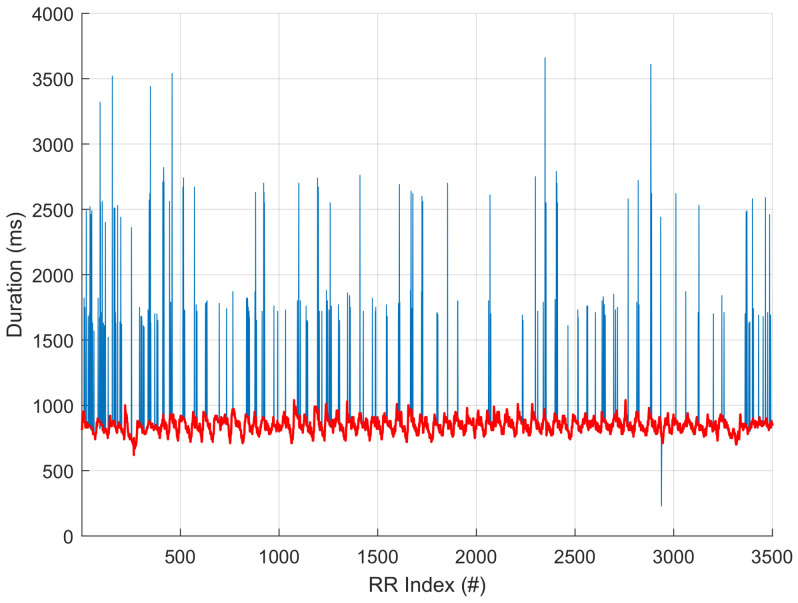
Input RR intervals (blue line) and output of the de-noising two-pass conditional median filter (red bold line).

The PhysioNet Apnea Challenge database is composed of 35 training nights (20 “A” apneic subjects, 5 “B” borderline subjects, 10 “C” non-apneic subjects) and 35 “X” test nights (for which the AHI was not initially disclosed). The hypnogram is not provided. We apply the sparse decomposition algorithm on the HR series extracted from these 70 nights using the “QRS” files, which are unaudited and contain measurement errors; these generally appear as spikes in the RR time series (see Figure 6). The target is the AHI, now known for these 70 nights.

The winner of this challenge achieved a score of 13,726 correctly classified 1-min time windows labelled as apnoea out of 17,268 (78.91%) [48]. To develop their model, they utilized the fraction of the signal power in the frequency range of 0.5 to 2.2 cycles per minute, computed over a five-minute time window. By applying a threshold to this relative power measure, they classified the center one-minute window as apnoea if this relative power exceeded the threshold. Consequently, their approach focused on detecting the recurring patterns of apnoea events rather than identifying individual events, which is the principle of the sparse method presented here.

To reduce the number of RR errors, we slightly process the extracted RR time series by a two-pass conditional median filtering. For both steps, we first detect the time instants for which the absolute value of the difference between the raw RR and the median filter output is greater than a predefined threshold. In such cases, we replace the RR value with the filter output. The median filter orders are 17 and 13, respectively, and the thresholds are 40 and 20. This is the only pre-processing step we perform before applying our algorithm.

As an illustration of the de-noising algorithm, the following figure depicts the result on night “X-25”. The blue curve represents the initial RR and the red one is the result.

## 3. Results

### 3.1. Evaluation of Internal Data

**Figure 7:**
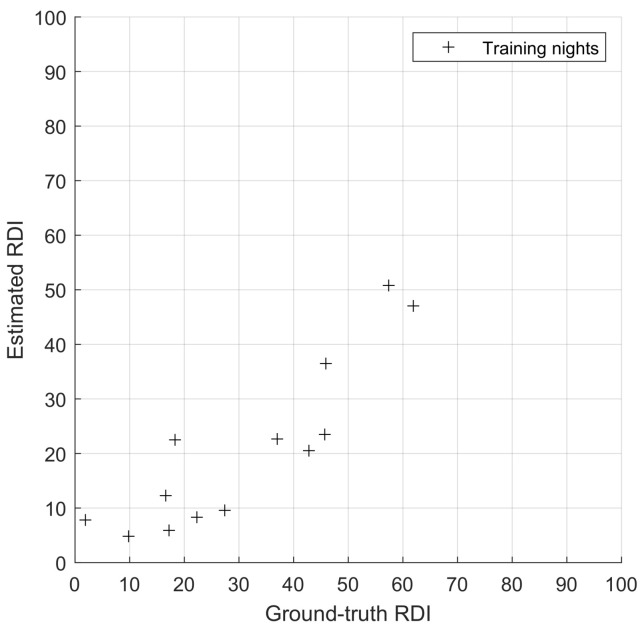
Results on the learning dataset, i.e., correlation of 0.88; *x*-axis: reference RDI; *y*-axis: estimator’s output.

In Figure 7, a comparison between ground-truth and estimated *RDI* is presented for the training nights (one night per recording). The results are promising, and although they would not allow for accurate categorisation of apnoea severity (i.e., on a four-class, standard assessment of severity), they are still a good prediction of the apneic status of a given person or recording.

**Figure 8:**
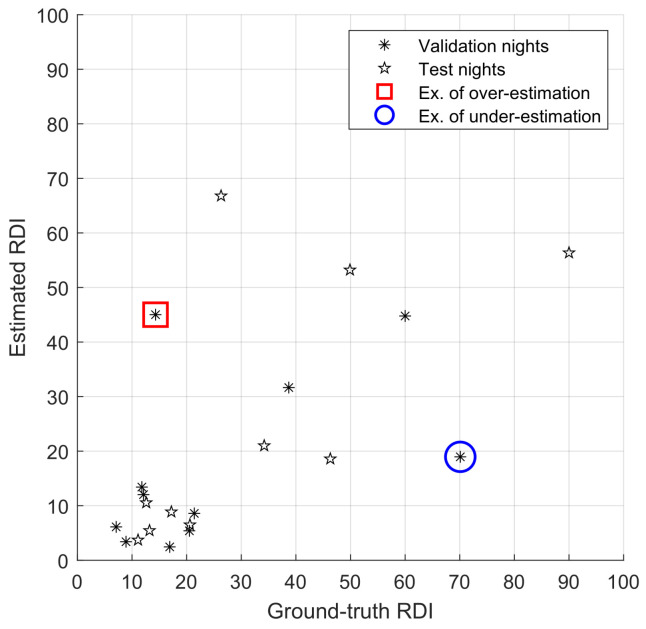
Results of the validation (black asterisks—correlation of 0.49) and on the test datasets (black stars—correlation of 0.66); we highlight a case of over-estimation (red square), as detailed in Figure 10, and of a case of low sensitivity (blue circle), as detailed in Figure 11; *x*-axis: reference RDI; *y*-axis: estimator’s output.

Moving forward on estimating performances on the validation and testing datasets (see Figure 8), several misestimation examples can be observed in under- and over-predictions.

**Figure 9:**
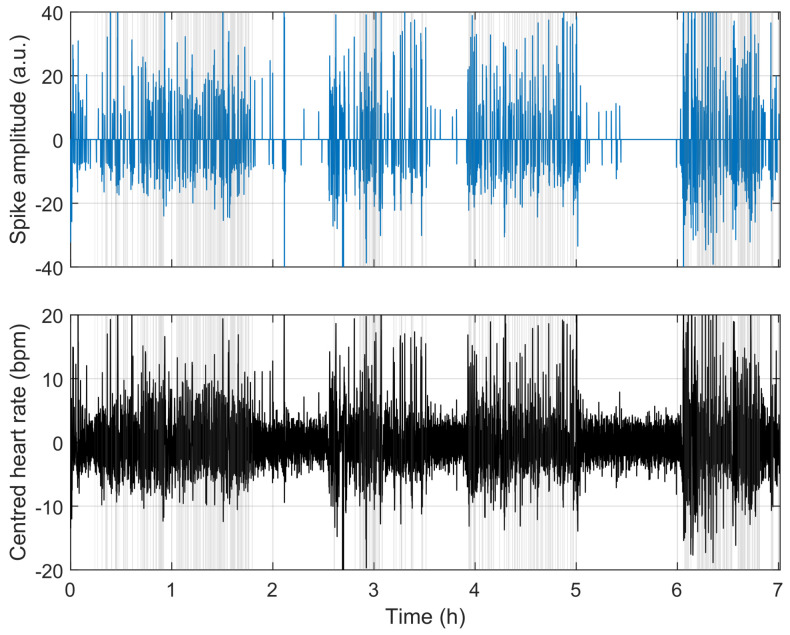
Example where the model works as intended for a whole night. The figure shows the output of the sparse model (upper blue spikes), the high-pass filtered heart rate (lower black curve), and the ground-truth apnoea (vertical grey patches).

**Figure 10:**
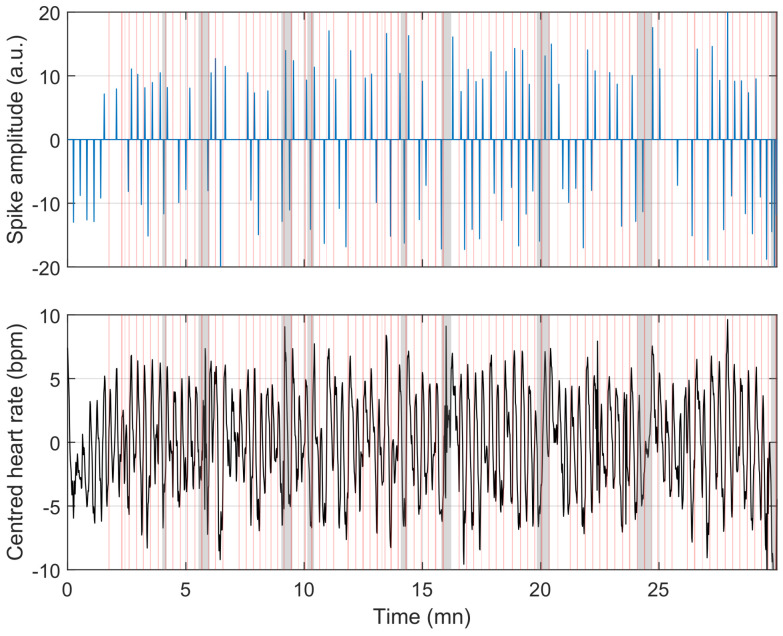
Example of over-detection using the same representation as in Figure 9; errors are explained by limb movement-induced cardiac responses (highlighted by the red patches). These have a similar heart rate profile to apnoea and are, therefore, detected as such.

**Figure 11:**
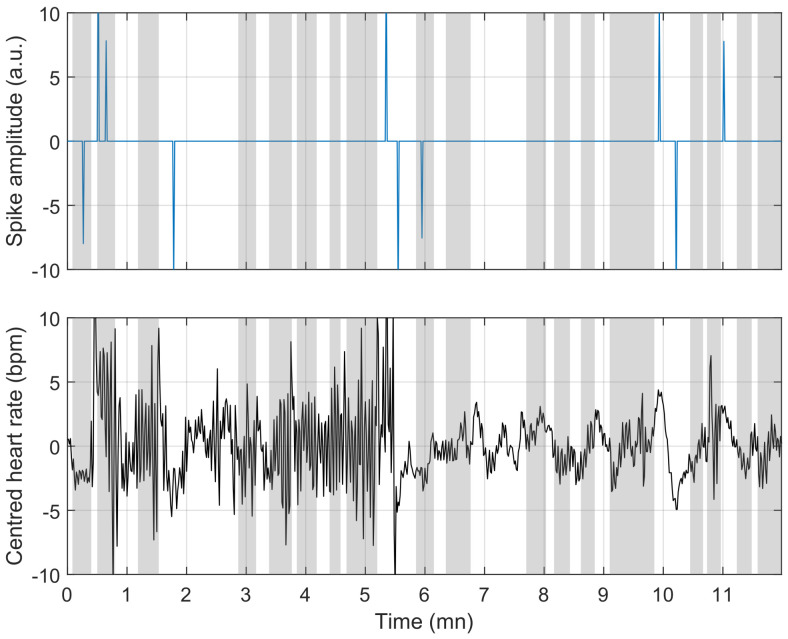
Same representation as in Figure 9 and Figure 10. Example of under-estimation; in the latter case, there seems to be no link between events in the heart rate and ground-truth apnoea.

To understand the reasons for misclassification, we compare the model’s behaviour in Figure 9, Figure 10 and Figure 11. Figure 9 shows a situation where the link between heart rate and apnoea is behaving as observed in training, while Figure 11 illustrates a situation of over-estimation, which will be described below (highlighted by the red square in Figure 8), and a situation of under-estimation (highlighted by the blue circle in Figure 8).

The figure above is representative of most situations. The two figures below represent challenges to the detection encountered in a few of our recordings. These events have effects similar to apnoea on the heart rate (here, limb movements), and a situation where there is apparently no link between the ground-truth apnoea and the heart rate. In both cases, nothing can be done without supplementary information.

Thus far, the results are encouraging, even though part of the recordings are problematic and would require either additional signals and/or careful screening (e.g., of limb movements). In the following subsection, we apply our method to the PhysioNet Apnea Detection Challenge data.

### 3.2. Evaluation of the PhysioNet Apnea ECG Database

In this paper, we focus on high *RDI* (or AHI) indexes as defined in Equation (5) by the AASM [45] using only the heart rate. Both standardised indexes do not take into account the duration of apnoea, which is used in the labelling of the PhysioNet data, as indicated below. However, comparing our results with those from the results of the PhysioNet Challenge competitors [49] (https://physionet.org/content/challenge-2000/1.0.0/, accessed on 10 November 2022) is not easy because of the differences between the performance criteria used.

Moreover, some competitors employed their own QRS detection algorithms and carried out significant signal processing to eliminate outliers and filter the signals, while others utilized an ECG-derived respiratory (EDR) signal, which is easier to obtain from the complete ECG (or PPG) [4,5] than from the 1Hz sampled HR (as in our case, as explained in the introduction) or specific waves of the QRS complex. For these reasons, it is difficult to provide an accurate comparison of performances. However, the usage of this dataset enabled us to evaluate our algorithm on a public database.

**Figure 12:**
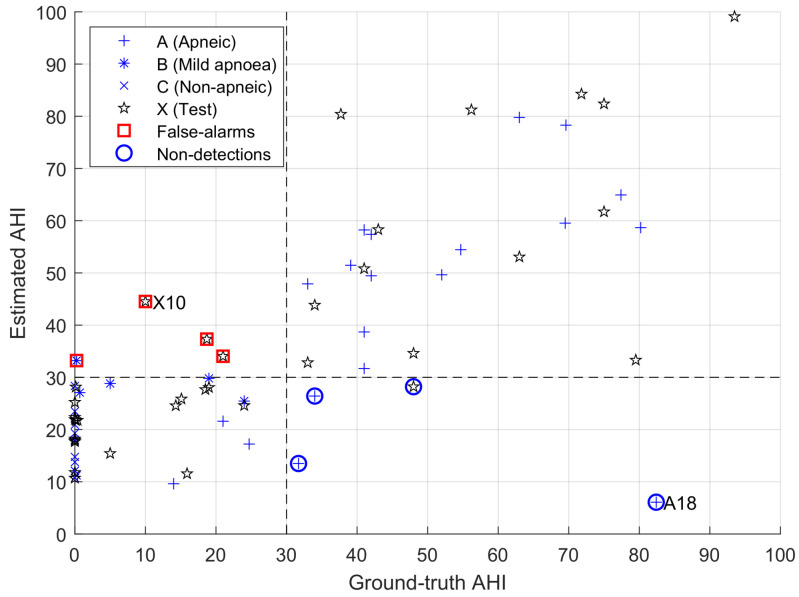
Method’s output on PhysioNet data.

From the de-noised RR signal, we compute the instantaneous heart rate, which we interpolate to resample at 1 Hz. We then apply the proposed sparse decomposition algorithm using a Gaussian window (σ=4) as the reference signal and a regularisation parameter of λ=300. Finally, we count only the number $N_{p}$ of positive coefficients and estimate the AHI as $\frac{N_{p}}{D}$, where *D* denotes the duration of the recording. As the hypnogram is unavailable, we cannot divide by the actual number of hours of sleep or remove false detections during “wake” hours. The results are shown in Figure 12.

The correlation between our estimate and the actual AHI equals 0.75. We also consider the detection of the apneic characteristic of a recording. Our objective is to restrain our method to the detection of severe apnoea. To achieve this, we utilise a threshold of 30 on the ground-truth AHI, represented by a vertical dashed line in Figure 12.

Recordings with an AHI value above 30 are considered severely apneic, while those below 30 are moderate-to-non-apneic. We use the same value of the threshold on the estimated AHI (represented by a horizontal dashed line) to evaluate the detection performance. We are now able to count the occurrences of correct and false detections. In this database, our results show a sensitivity of 0.87 (27 out of 31 severely apneic recordings are correctly detected) and a specificity of 0.90 (only 4 out of 39 moderate-to-non-apneic recordings are incorrectly classified as severely apneic).

**Figure 13:**
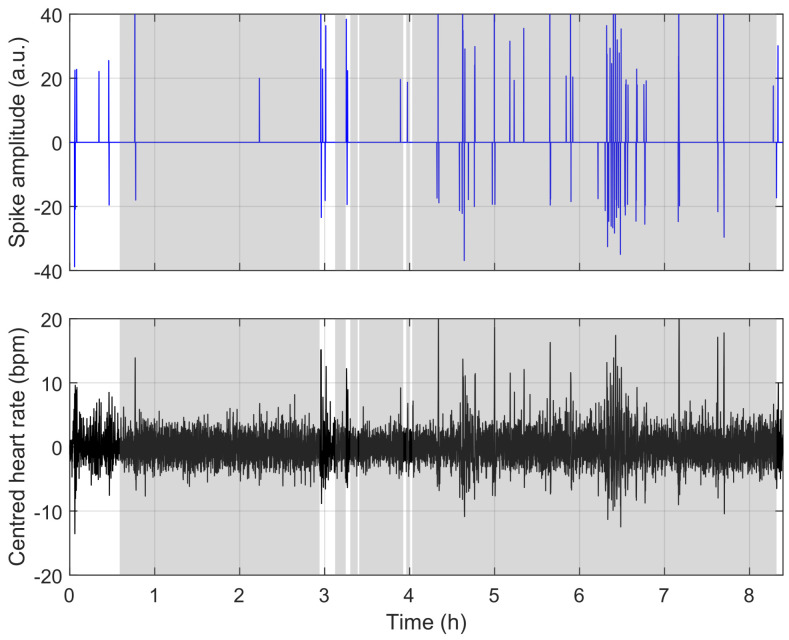
PhysioNet: recording “A-18”, an example where our model underestimates AHI. The same representation as in Figure 9; there is no visible bradycardia–tachycardia event associated with apnoea (which constitutes most of the recording, according to the ground truth).

**Figure 14:**
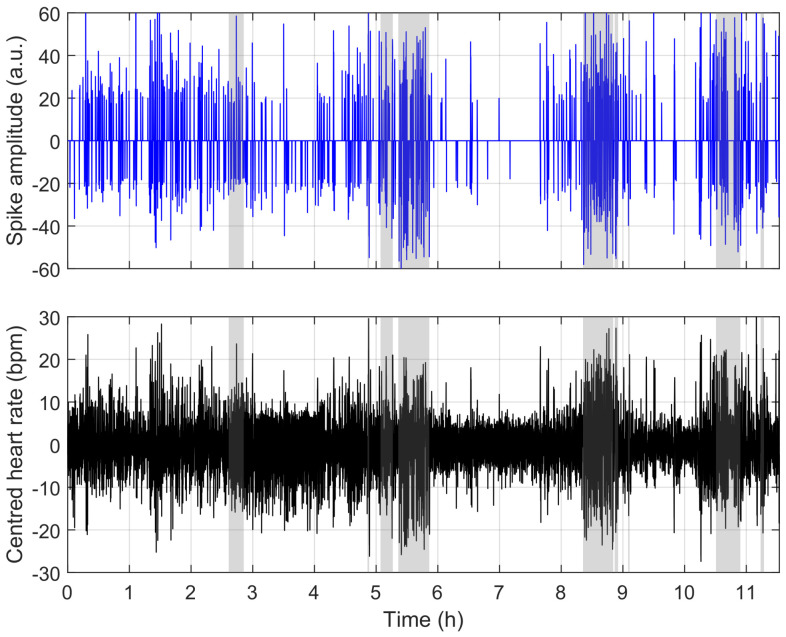
PhysioNet: recording “X-10”, an example where our model over-estimates AHI. The same representation as in Figure 9; the heart rate variability is high for this recording; segments of the signal may resemble bradycardia–tachycardia, inducing an overestimation of apnoea while making true events less visible.

**Figure 15:**
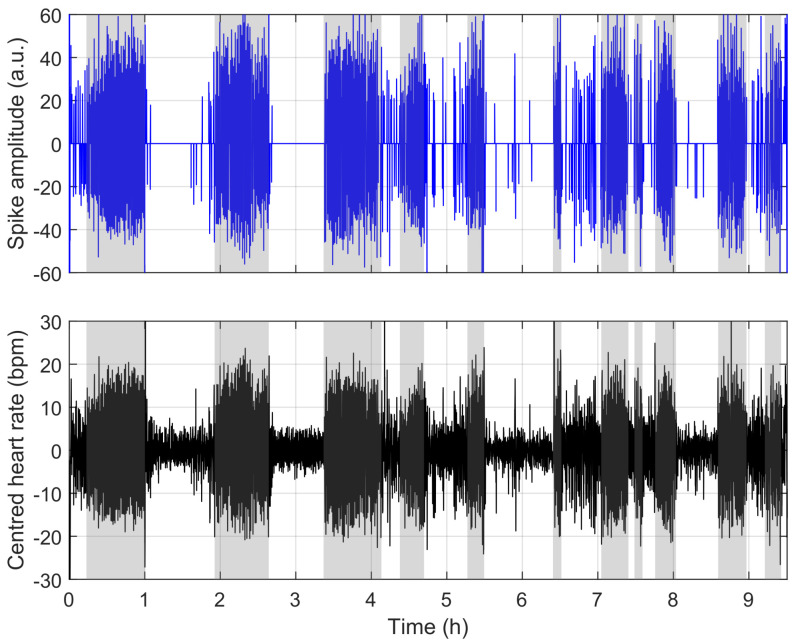
PhysioNet: recording “A-03”, an example where our model works as expected. The same representation as in Figure 9. In this recording, bradycardia–tachycardia associated with apnoea appear clearly on the sparse decomposition, allowing for a very good detection of apnoea.

**Figure 16:**
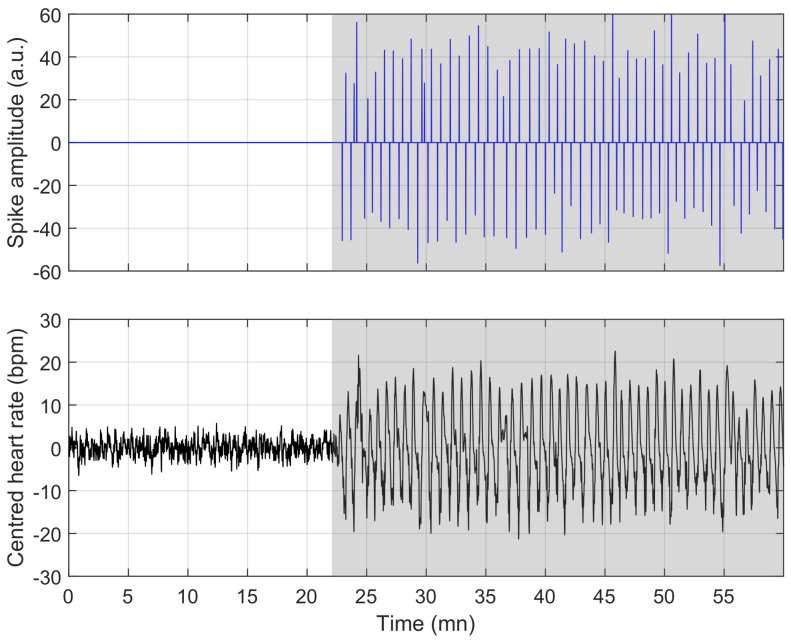
PhysioNet: magnified section of recording “A-03”, extracted from Figure 15. The difference between non-apnoea and apnoea appears clearly and is perfectly captured by the sparse decomposition, while an energy detector should work in such a simple case.

Here again, a few nights are located away from the diagonal. Using the aforementioned thresholds, we highlight cases of over-estimation (red squares) and under-estimation (blue circles). In the following, we focus on recording “A-18”, a case of under-estimation for which the presence of apnoea does not induce visible bradycardia-tachycardia events in the heart rate (see Figure 13), and recording “X-10”, where high heart rate variability generates false positives while making the bradycardia-tachycardia events hardly visible (see Figure 14). An example where the model works almost perfectly is illustrated in Figure 15 and Figure 16, which correspond to recording “A-03”, the whole night, and a zoom-in of the time period between hour 3 and hour 4, respectively.

These observations strengthen the conclusions drawn from our data. The heart rate is not an ideal choice for apnoea detection, due to the many variabilities between-and-within recorded subjects; however, it often allows a first estimation of a subject’s apnoea severity, which could serve as a preliminary screening technique, to be confirmed in further steps (expert evaluation, screening through questionnaires).

## 4. Discussion

The results of our study are promising, but not sufficient for accurately evaluating the Apnea-Hypopnea Index (AHI) for standard four-class classification of apnoea severity. Nevertheless, we find these results encouraging, particularly on our internal database, where we have access to accurate apnoea scoring from respiratory signals and a hypnogram. This enables us to select an adaptive detection threshold computed from the observed heart rate on each night/subject to address the high inter-individual variability. Additionally, we identified detection problems induced by cardiac events that resemble apnoea but result from other pathologies.

In the PhysioNet database, we only used the RR extracted from the available full ECG and processed only the RR time series to remove the simplest artefacts. Furthermore, we were unable to select an adapted threshold due to the unavailability of the hypnogram. This partly explains the difference in observed performance. However, our results still appear coherent with the heart rate’s sparse description. Our performance is likely lower than the best obtained in the PhysioNet Apnea Challenge, as we only utilised the RR/HR signal, which was our objective, unlike competitors who used the whole ECG signal.

## 5. Conclusions

In this paper, we considered the application of Bernoulli–Gaussian sparse decomposition on the heart rate signal, which provides an elegant graphical visualisation of the succession of bradycardia and tachycardia events that appear in apneic subjects.

We focused on the detection of apnoea (and related respiratory events) and the estimation of associated indexes (AHI and/or RDI) as an obvious candidate example. Our results on both our internal database and the PhysioNet database are encouraging, although a few recordings are poorly assessed. Firstly, there is a specificity problem associated with other causes for cardiac events (e.g., limb movements) that cannot be discriminated against based solely on the heart rate. Secondly, the performance of the estimator is strongly impacted by data quality. Thirdly, there is significant inter- and intra-variability among subjects, making it a challenge to tune an efficient *RDI* or AHI estimate on all kinds of subjects and recordings. Finally, even in the best-case scenario, the estimated indexes are not accurate enough to precisely assess clinical apnoea severity. Depending on the objectives of a study, this approach may not be considered a valid alternative to questionnaires and other gold-standard methods of assessment. Furthermore, while this approach may not be performant enough to stand alone as an *RDI* estimator, it could be a valuable addition as an input in decision-combination algorithms.

Perspectives of this research include considering the beginning and end of the bradycardia detection window as parameters to be tuned to improve performance, as well as incorporating additional signals to reduce ambiguity, allowing to distinguish between apnoea and other events with similar effects that may impact heart rate (e.g., limb movements). The most important perspective of this work will involve using a coupled bradycardia–tachycardia event as the reference input, which would require a positivity constraint on the sparse representation coefficients. This would require a total rework of the $L_{2}-L_{0}$ optimisation algorithm.

## Figures and Tables

**Table 1:** Dataset compositions in terms of apnoea severity, based on ground-truth *RDI*: non-apneic (*RDI* <5), mildly apneic (∈[5;15[), moderately apneic (∈[15;30[), and severely apneic (≥30).

Dataset	Non-Apneic	Mild	Moderate	Severe
Learning	1	1	5	6
(13 subjects)				
Validation	0	5	3	3
(11 subjects)				
Test	0	3	3	4
(10 subjects)				

**Table 2:** Dataset compositions in terms of sex, age, and body mass index (BMI).

Dataset	Female/Male	Age	BMI
Learning	3F/10M	53.8±11.8	30.0±6.5
(13 subjects)		(from 35 to 70)	(from 20.9 to 41.4)
Validation	4F/7M	55.5±13.1	27.7±4.2
(11 subjects)		(from 35 to 77)	(from 21.2 to 35.9)
Test	3F/7M	52.0±14.6	27.9±5.9
(10 subjects)		(from 36 to 71)	(from 20.5 to 37.0)

## Data Availability

The development data, which the model was tuned on, are private. Supplementary data, on which the model has been further tested, come from the PhysioNet Apnea Detection Challenge (https://www.physionet.org/content/apnea-ecg/1.0.0/, accessed on 10 November 2022). We would like to thank PhysioNet for making their data available.
